# Modified Split Ring Resonators Sensor for Accurate Complex Permittivity Measurements of Solid Dielectrics

**DOI:** 10.3390/s20236855

**Published:** 2020-11-30

**Authors:** Amer Abbood al-Behadili, Iulia Andreea Mocanu, Norocel Codreanu, Mihaela Pantazica

**Affiliations:** 1 Department of Telecommunication, Telecommunications and Information Technology, Faculty of Electronics, University POLITEHNICA of Bucharest, 060042 Bucharest, Romania; amer_osman@uomustansiriyah.edu.iq; 2 Electrical Engineering, College of Engineering, Mustansiriyah University, Baghdad 00964, Iraq; 3 Center for Technological Electronics and Interconnection Techniques, Department of Electronics Technology and Reliability, Telecommunications and Information Technology, Faculty of Electronics, University POLITEHNICA of Bucharest, 060042 Bucharest, Romania; norocel.codreanu@cetti.ro (N.C.); mihaela.pantazica@cetti.ro (M.P.)

**Keywords:** metamaterials, planar sensor, non-invasive, Split Ring Resonator, dielectrics measurements, RF absorbing materials

## Abstract

In this paper, a sensor using modified Split Ring Resonators (SRRs) is designed, simulated, fabricated, and used for advanced investigation and precise measurements of the real part and imaginary part solid dielectrics’ permittivity. Adding vertical strips tightly coupled to the outer ring of the SRR leads to the appearance of two resonant frequencies at 1.24 GHz and 2.08 GHz. This modified geometry also assures an improved sensitivity. Using the full wave electromagnetic solver, both the unloaded and loaded sensors are investigated. The numerical simulations are used to develop a mathematical model based on a curve fitting tool for both resonant frequencies, allowing to obtain analytical relations for real and imaginary parts of permittivity as a function of the sample’s thickness and quality factor. The sensor is designed and fabricated on 1.6 mm thick FR-4 substrate. The measurements of different samples, such as transparent glass, acrylic glass, plexiglass, and Teflon, confirm that the modified SRR sensor is easy to implement and gives accurate results for all cases, with measurement errors smaller than 4.5%. In addition, the measurements highlight the importance of the second resonant frequency in the cases in which numerical limitations do not allow the usage of the first resonant frequency (1 mm thick sample).

## 1. Introduction

The electric complex permittivity is one of the most important parameters of material characterization. It is utilized in a large range of applications such as: Material description [1,2], tests of organic tissue [3,4] microfluidics [5,6,7,8], bio sensing [9,10,11], ecological operators [12,13], and quality control in the food industry [14,15]. Accurate determination of the permittivity is an important task and a great challenge for microwave engineering, in general, and therefore many solutions have been investigated lately.

A relatively new option for implementing sensitive planar sensors is to use metamaterials. Metamaterial structures present a major advantage over other conventional options: They can be artificially tailored to achieve better resolution and accuracy. In the last few years, an increased interest for studying the sensors based on resonant metamaterial structures such as Split Ring Resonator (SRR) and Complementary Split Ring Resonator (CSRR) has been noticed due to minimal efforts in sample preparation, nondestructive effect, ability to characterize both low and high losses materials, and higher sensitivity [2,16].

Considering these important advantages, we present a modified SRR sensor for accurate complex permittivity of solid dielectrics. It is implemented in planar technology and it exhibits two resonant frequencies, which are used to overcome numerical limitations that may appear in real-life measurements. The results obtained for our sensor are compared with similar approaches existing in the literature.

There are many SRRs sensors depending on a single resonant frequency, which is produced by the related resonator circuit, and the main focus of the authors is only on the detection of the real part of permittivity.

For example, in reference [5], an SRR sensor working at 2 GHz is proposed. It is implemented in planar technology, on Rogers AD1000 substrate, and it is used to measure the thickness of thin films, as well as the electric permittivity for both dielectrics and liquids, being fully submersible. The extraction of the imaginary part of the permittivity is not rigorous as the authors conclude [5]. They also argue that a more thorough and systematic study investigating many more combinations of real and imaginary parts of the permittivity [5] should be considered for further improvements. Nevertheless, the authors suggest that another version of the sensor should be developed to obtain two resonance frequencies, in order to determine the complex permittivity and the thickness of solids at the same time [5]. The sensor we propose has two resonant frequencies able to measure complex permittivity with errors smaller than 4.5% and for samples with thicknesses from 1 mm to 10 mm.

In ref. [16], an interdigital capacitor based SRR (IDC-SRR) sensor for dielectric testing is investigated. The authors also propose and analyze a meandered line based split ring resonator (ML-SRR) RF sensor for magnetic testing. Both sensors work at 2.45 GHz and are implemented on RT/Duroid 6006 substrate. The accuracy of the real part of permittivity measurements is more than 94% [16], but still, these sensors are not able to measure the complex permittivity as our proposed sensor. Our sensor can measure the imaginary part of permittivity with comparable accuracy.

Another SRR based sensor for magnetodielectric substrates characterization is the one presented in [17]. The device is fabricated using the microstrip technology on a 1.27mm-thick RT/Duroid 6006 substrate and working at 2.5 GHz [17]. The SRRs are magnetically coupled to the microstrip line allowing both electric permittivity and magnetic permeability measurements. Still, only the real parts are measured. Our sensor can measure both real and imaginary parts for the complex permittivity and the errors for real part measurements are comparable for the same samples as in [17].

A different approach for the sensor design is presented in [18]. It uses a two-layer and three-layer magnetic coupled SRR for higher sensitivity, better stability, and stronger anti-jamming ability from the external interface. These sensors have dimensions of 0.052λ × 0.052λ allowing miniaturization, but no imaginary parts of permittivity measurements are carried out [18]. The influence of the thickness of the sample is not considered in this study. On the other hand, the errors for real part of permittivity measurements are similar to ours.

Another modified SRR sensor is presented in [19], but it is used for thin-film detection, not for thicker dielectrics as ours. Furthermore, for this thin-film sensor, only the frequency shifts are investigated [19], without determining the complex permittivity of the MUT as in our case.

In [6], an SRR-based sensor is presented for measurement of complex permittivity of liquids. The sensitivity of the sensor is improved by overlapping the middle part of the outer ring of the SRR and part of the feeding line. It is best suited for measuring mixed liquids and determining the complex permittivity for each component, but no study regarding its application for solid materials characterization is done.

Regarding the characterization of material under test (MUT), several techniques have been proposed and employed for the permittivity. The most important ones can be categorized as free-space methods, transmission-line methods, and resonant cavity methods [20].

The free-space method commonly employs using extremely directive lens and horn antennas placed on both sides of the MUT. The vector network analyzer (VNA) is connected to the antennas to measure the scattering parameters and phase constant to characterize the sample [21]. This technique has the advantage of being contactless and not wasteful, but it demands the usage of expensive lenses and horn antennas, as well as the need for a large sample.

Another technique for measuring the material’s electric permittivity is the transmission-line method. In this method, the MUT is used as a loading material for transmission lines, such as a slice of material that can be incorporated to a waveguide [22] or the deposing materials of a coaxial line that can be replaced by the MUT [23]. The scattering parameters from the MUT-filled region provide the data necessary to extract the material’s properties. This method is comparatively lower cost than the free-space method. However, the sensitivity of the scattering parameters approach is not very efficient for low loss samples, and the sample elaboration is also very often a challenging task [24]. The structures of microstrip-line and stripline are also used for this technique [25].

One quite accurate technique is the resonant cavity method [26]. In this method, a cavity resonator is loaded with the MUT, and the shift in the resonance frequency and the variation in the quality factor are determined. Circular resonators and microstrip-line resonators have been also employed for this purpose [27] other than a conventional box resonator. This technique also needs accurate sample elaboration.

In order to overcome the limitations described above and to obtain all the information required to accurately characterize the complex electrical permittivity of a solid material, we propose a modified SRRs planar sensor for noninvasive complex permittivity measurements of solid materials. The resonant structures studied in this paper are based on the well-known SRR structures, with a modified topology, improving the selectivity and assuring two resonant frequencies. The measurement technique adopted in our research is the resonant cavity method.

The sensor is implemented on FR-4 substrate, offering portability, low-cost manufacturing, and easy-to-use and easy-to-interpret results. The structure is designed to be easily manufactured on a single metal layer, while allowing the easiness of integration of resonator elements at the same level.

The propagation phenomena occurring in these modified resonant structures can also be used to create RF absorbing materials, which can lead to designing efficient microwave absorbers for different applications, such as 5G antennas and automotives.

## 2. Modified SRR Sensor Design

### 2.1. Resonant Structures for Higher Selectivity

**Figure 1:**
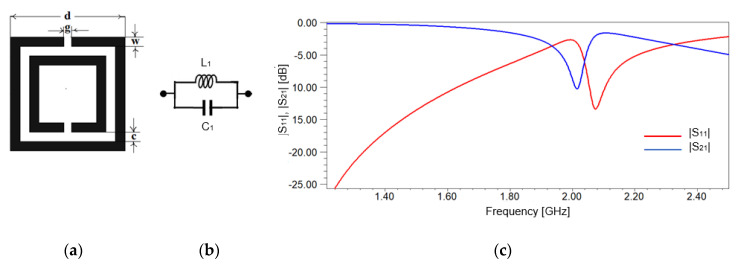
Split Ring Resonator: (**a**) Split Ring Resonator (SRR) with physical dimensions; (**b**) equivalent circuit; (**c**) frequency response of the scattering parameters for SRR.

In 1999, J. Pendry proposed a motivating sub wavelength element defined as split ring resonator (SRR) to realize negative permeability [28]. The SRR structure consists of two highly closed concentric metallic split ring resonators etched on a substrate, with two gaps orientated in opposite directions, as shown in Figure 1a. When a magnetic field perpendicular to the ring surface is applied, a current is induced through the rings. These currents go from one ring to another due to the distributed capacitance that appears between them.

The goal of this work is to create an affordable, low-cost manufacturing sensor. Therefore, we choose a FR-4 substrate with relative permittivity ε_r_ = 4.4 and the dissipation factor, tan δ, approx. 0.02. The thickness of the substrate is equal to 1.6 mm and the cooper metallization electrodeposited on both sides of the substrate has a thickness of 18 µm. Another goal of the work is to characterize materials in the low-GHz band. So, the SRR’s dimensions are considered to have measuring applications for frequencies around 2 GHz. In this case, the distance between the rings is c = 1.52 mm, the width of the rings is w = 1.52 mm, the width of the gap is g = 1.22 mm, and the length of the external ring is d = 18.4 mm. Figure 1a shows the geometrical design of the SRR cell.

The material characterization technique used to determine dielectric properties is the resonant one. This process monitors the frequency shift and the variation of the quality factor due to MUT loading the resonator, which is currently represented by the SRRs elements.

The equivalent circuit of the classical SRR in Figure 1a represents a resonant cavity modeled by a LC circuit, Figure 1b, where the inductance *L*_1_ models the effect of the conductive strips of the rings and the capacitance *C*_1_ models the effect of the gap between the two rings [29]. The values for the inductance *L*_1_, the capacitance *C*_1_ and the gap capacitance appearing at the end of each ring, *C_g_* are computed based on the geometrical dimensions of the SRR, the substrate’s properties, and the relations given in [29]: *L*_1_ = 38.58 nH, *C*_1_ = 152.49 fF, *C_g_* = 1.69 fF. The gap capacitance, *C_g_*, can be neglected in comparison to the value of the capacitance *C*_1_ [29].

In this case, the total impedance of the resonant equivalent circuit can be written:

(1) $$Z_{T,1}=\frac{j\omega L_{1}}{1-\omega^{2}L_{1}C_{1}}$$

and the resonant frequency is [29]:

(2) $$f_{r,1}=\frac{1}{2\pi \sqrt{L_{1}C_{1}}}=\;2.075\;\mathrm{GHz}$$

The resonant frequency read from Figure 1c is 2.08 GHz, in very good agreement with the one computed using (2). Additionally, Figure 1c shows that the resonant frequency is not so well emphasized, without a sharp response of the SRR.

**Figure 2:**
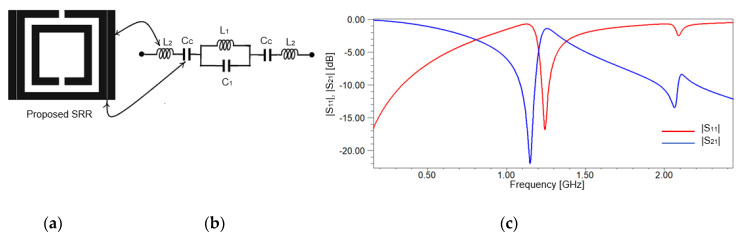
Proposed SRR: (**a**) SRR with vertical strips, placed at the distance s from the initial SRR in Figure 1a; (**b**) equivalent circuit; (**c**) frequency response of the scattering parameters for proposed SRR.

To obtain a better selectivity, the classical SRR depicted in Figure 1a is modified by adding microstrip vertical strips (VS) of width, w, leaving a gap, s, between the SRR and the vertical strips, as presented in Figure 2a. The gap is set to 0.2 mm to assure a tight coupling effect, but also considering the technological restrains. The dimensions of the resonant structures presented in Figure 1a or Figure 2a are given in Table 1.

The substrate used for simulations in both figures is FR-4, with a thickness of 1.6 mm and the relative electric permittivity of 4.4. The equivalent circuit for the modified SRR proposed in Figure 2a is the one in Figure 2b, where the effect of the strips is modeled by the inductance *L*_2_ and the coupling effect is modeled by the capacitance *C_C_*. Using our geometrical dimensions and the relations from [29], we obtain: *L*_2_ = 11.04 nH and *C_c_* = 1.49 pF.

Regarding the strong couplings between the vertical strips and the rings of the SRR, the strips themselves lead to the appearance of a second resonant frequency, which assures an improvement in resolution, as shown in Figure 2c, compared to the frequency response from Figure 1c.

The appearance of the second resonant frequency in Figure 2c can be explained by computing the impedance of the resonant equivalent circuit in Figure 2b:

(3) $$Z_{T,2}=\frac{2(1-\omega^{2}L_{2}C_{C})}{j\omega C_{C}}+\frac{j\omega L_{1}}{1-\omega^{2}L_{1}C_{1}}$$

Considering the first resonant frequency given by relation (1), we can rewrite relation (3):

(4) $$Z_{T,2}=\frac{2\left[1-{\left(\frac{\omega }{\omega {’}_{r,1}}\right)}^{2}\right]}{j\omega C_{C}}+\frac{j\omega L_{1}}{1-{\left(\frac{\omega }{\omega_{r,1}}\right)}^{2}}$$

where $\omega {’}_{r,1}=2\pi f{’}_{r,1}$ and

(5) $$f{’}_{r,1}=\frac{1}{2\pi \sqrt{L_{2}C_{C}}}$$

Using the values determined previously for *L*_2_ and *C_C_* and using relation (5), we can compute the second resonant frequency equal to 1.23 GHz. From Figure 2c, we read that the second resonant frequency is 1.24 GHz. So, the equivalent circuit for the SRR in Figure 1b and for the proposed SRR in Figure 2b used to compute analytically the resonant frequencies proves an accurate modeling of the resonant structures. In addition, we further consider analysis for only the *S*_21_ parameter because it is more sensitive than *S*_11_. The two resonant frequencies that occur can be used to obtain a better resolution for measurements. Furthermore, because of an increase in the equivalent capacitance due to adding vertical strips, the resonant frequency decreases to smaller values, around 1.24 GHz, as one can observe in Figure 2c. The other resonant frequency remains the same as in Figure 1c, around 2 GHz.

The next step is to add access transmission lines and practically transform the structure into a planar sensor made of Vertical Strips Split Ring Resonators (VS-SRRs). We calculate the width of the access transmission line corresponding to 50 Ω to be 6.166 mm.

**Figure 3:**
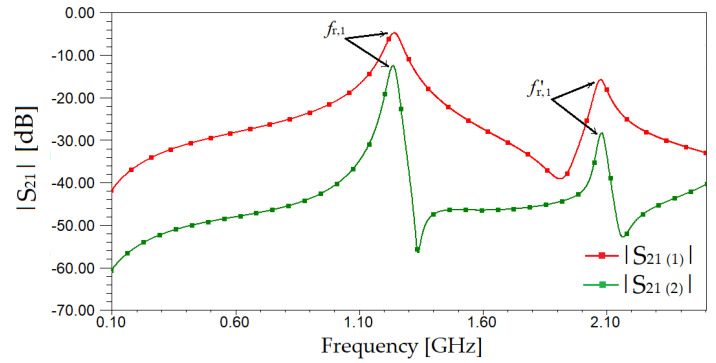
Scattering parameters for sensors made of one modified SRR-S_21(1)_ and two modified SRRs-S_21(2)_.

Next, two cases are analyzed: The sensor containing one modified SRR cell and the sensor with two modified SRRs cells. The geometrical dimensions for the SRRs and the vertical strips are the ones given in Table 1. Additionally, we keep the same substrate as in our previous analysis. The overall dimensions of the sensor, including the access transmission lines, are 0.5λ × 0.16λ for one-cell sensor and 0.7λ × 0.16λ, respectively, for the two-cell sensor. Still, the sensors can be easily rescaled and re-designed to be used for other frequency applications or for further miniaturization. The frequency response of both sensors is given in Figure 3.

As it can be observed in Figure 3, selectivity has increased due to adding access transmission lines and increasing the number of cells. In addition, one can observe that both resonant frequencies, *f*_r,__1_ and *f’*_r,__1_ are well emphasized and can be used for further computations. Adding a new modified SRR has led to a better selectivity than using only one. This indicates an improvement in the quality factor of the sensor and thus it will provide a better accuracy for characterizing the dielectric constant of the samples. We consider the results obtained for the two cells vertical strips SRR (VS-SRR) are good enough to further investigate this sensor and not add more SRRs and complicate the structure or increase the manufacturing cost.

### 2.2. The Resonant Frequencies Analysis

**Figure 4:**
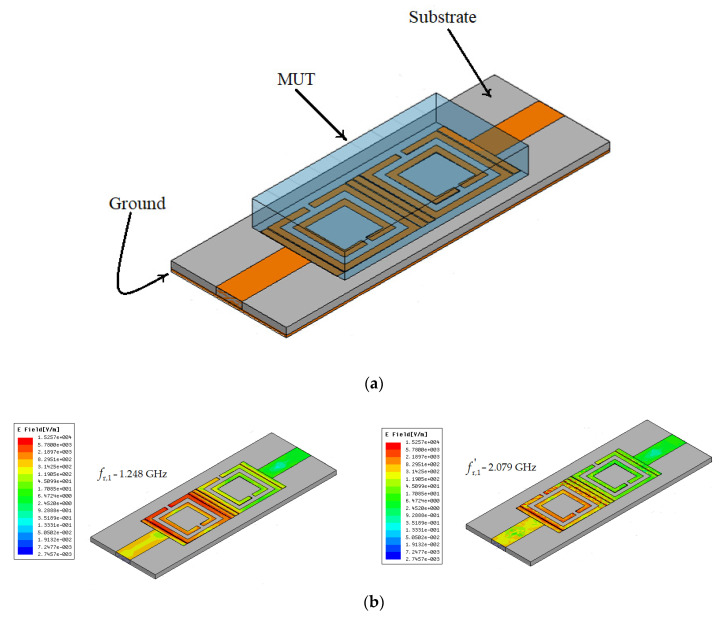
The vertical strips (VS)-SRR sensor: (**a**) Setup of the VS-SRR sensor with material under test (MUT) placed over the two modified SRR unit cells, (**b**) 3D representation of the intensity of the electric field at the first resonant frequency and at the second resonant frequency.

The material under test (transparent box) is placed on the SRR unit cells of the VS-SRR sensor, as depicted in Figure 4a, covering the whole area of the sensor for having an efficient perturbation of the *E*-field and assuring the resonance frequency shift required for precise measurements. When the resonance occurs, the total electric field will be confined to a smaller region of split ring resonator, where the sample is usually placed as shown. This confined electric field is capable of sensing an even smaller change in the dielectric constant of the test sample. The response of the microwave sensor to the change in the effective dielectric constant of the surrounding can be noticed in terms of the change in resonant frequency and the quality factor of the loaded structure [5]. The intensity of the electric field through the sensor, analyzed at the two resonant frequencies, is presented in Figure 4b.

The results in Figure 4b show that when the resonances occur, the total electric field is confined mainly to the first VS-SRR cell of the split ring resonator. This confined electric field is capable of sensing small changes in the dielectric constant of the MUT placed above the sensor. The response of the microwave sensor to the change in the effective dielectric constant is observed as a change in the resonant frequency and the quality factor of the loaded structure. Furthermore, in Figure 4b, one can observe the impact on the electric field distribution of adding vertical strips near the classical SRR. Introducing vertical strips near the SRR, as depicted in Figure 2a, basically increases the effective capacitance of the whole structure. This leads to higher electric field intensity in a small sensing region and, thus, obtaining an improved sensitivity of the sensor.

Moreover, we can see that the electric field is mainly concentrated in the first VS-SRR cell for both frequencies, but through capacitive coupling it propagates to the second cell as well. So, when using the MUT, it is important to place it on the whole sensor, to cover the whole sensing area made of both VS-SRRs.

**Figure 5:**
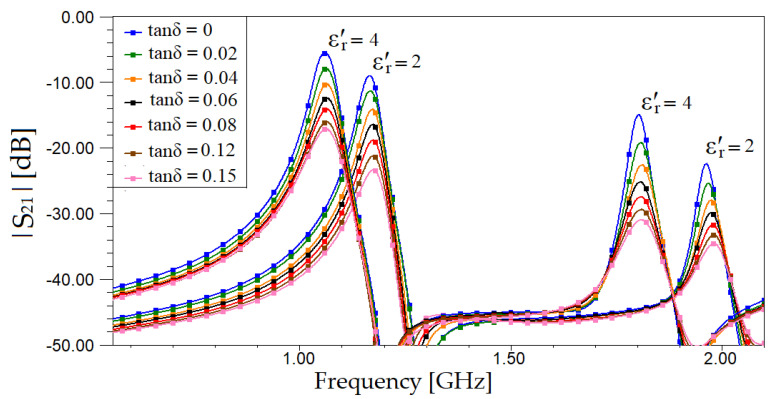
Variation of *S*_21_ (dB) response of the VS-SRR sensor with loss tangent value varying from 0 to 0.15 and for values of the relative electric permittivity, *ε*ʹ_r_ equal to 2 and 4.

Next, through full wave electromagnetic simulation in Ansys HFSS, we investigate how the resonant frequencies shift when loading the sensor with different solid dielectrics considered as MUTs. The resonant frequencies in Figure 3 are considered the reference ones for the unloaded sensor (*f*_r,1_, and *f* ʹ_r,1_). The proposed sensor is then loaded with various dielectric materials as MUT, with the real part of the relative electric permittivity, *ε*ʹ_r_ equal to 2 and to 4 and the loss tangent, tan δ, ranging from 0 to 0.15. The simulated transmission coefficient, *S*_21_ is depicted in Figure 5.

From Figure 5, it can be noticed that the shift for the resonant frequency *fʹ*_r,1_ is greater than the shift for *f*_r,1_ in the same conditions: Same variations of *ε*ʹ_r_ and tan δ of the material under test. In order to evaluate the sensitivity performance of both resonant frequencies based on the results in Figure 5, a relative frequency shift is defined as:

∆*f*_r_ = unloaded(*f*_r_) − loaded(*f*_r_) (6)

**Figure 6:**
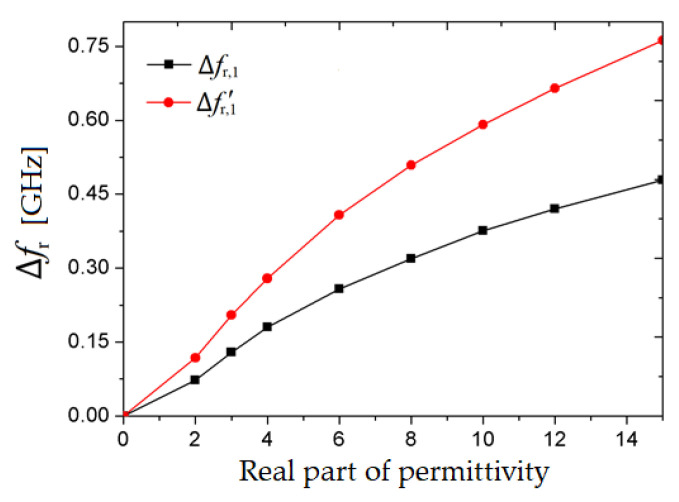
The sensitivity for both resonant frequencies, *f*_r,1_ and *f* ʹ_r,1_.

For our analysis, we consider a broad range of values for the real part of the permittivity, between 0 and 14, and investigate the relative frequency shift for both resonant frequencies. The results of the frequency shift variation, ∆*f*_r_ with respect to the real part of the relative electric permittivity for both resonant frequencies, are plotted in Figure 6.

From Figure 6, it can be noticed that the relative frequency shift corresponding to the second resonant frequency, *f*ʹ_r,1_ is greater than the relative frequency shift produced by the first resonant frequency, *f*_r,1_. This means that using *f*ʹ_r,1_ is considered a better option to obtain a higher sensitivity than using the first resonance frequency, *f*_r,1_. However, in the current work, both resonant frequencies of the VS-SRR sensor will be utilized for MUT characterization in order to add a higher degree of precision, especially if limited by technological or numerical constrains, as proved later when having real measurements.

## 3. Numerical Analysis

In our further numerical analysis, we investigate both resonant frequencies as argued above. For each of them, we consider the data obtained after full wave electromagnetic simulation, and using a curve fitting tool, we determine analytical expressions for both the real and imaginary parts of the permittivity. These are expressed as a function of the resonant frequencies, the MUT’s thickness, and the quality factor of the loaded sensor. A curve fitting tool is often used in the literature [1,16,17] for successfully estimating numerical expressions based on collected data from simulation or measurements.

In the unloaded situation, the simulated resonant frequencies (*f*_r,1_ and *f’*_r,1_) of the VS-SRR sensor are 1.24 GHz and 2.08 GHz.

Knowing that the quality factor for general resonators, Q can be written [30]:

(7) $$Q=\frac{f_{r}}{\Delta f}$$

where *fr* is the resonant frequency and *∆f* represents the relative 3dB bandwidth of the resonator’s frequency response; we determine the quality factors corresponding to the two resonances as being equal to 35.4 and to 65, respectively.

After loading the sensor with the material under test, a shift in the resonant frequency as well as a change in the magnitude of S_21_ (dB) are recorded as mentioned earlier in Figure 5. The values for the resonant frequencies (*f*_r,1_, *fʹ*_r,1_) and the quality factors are calculated from the response of the transmission coefficients and are then used to achieve a numerical expression with the aid of a curve fitting tool. A commercially available software OriginPro 8 [16] is used as a curve fitting tool for the data obtained after full wave electromagnetic simulation in Ansys HFSS. The equations are generated using the sets of obtained data. A particular profile curve is chosen based on the least error between the chosen profile and the sets of numerically obtained data [30], as it will be presented in the next sections.

### 3.1. Deduction of the Real Part of the Permittivity

To determine the type of dependency between the resonant frequency and the real part of the permittivity, we consider the expression for the resonant frequency [31]:

(8) $$f_{r}=\frac{1}{2\pi \sqrt{L(C+C_{load}})}$$

The capacitance introduced by the load, *C_load_* depends directly proportional to the real part of the electrical permittivity [31], so considering relation (8) as well, we obtain *f*^−2^_r_ ∝ *ε*ʹ_r_.

**Figure 7:**
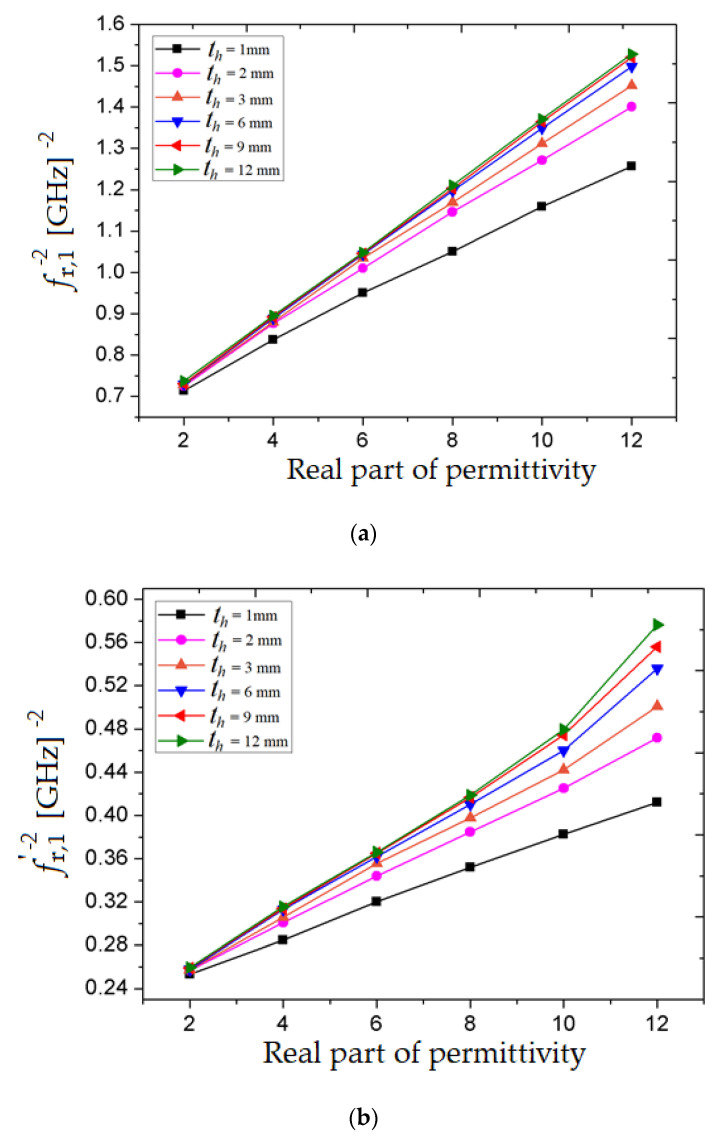
Resonant frequencies in terms of real permittivity (*ε*ʹ_r_) for different thickness of MUT: (**a**) First resonant frequency (*f*_r,1_)^−2^; (**b**) second resonant frequency (*f* ʹ_r,1_)^−2^.

So, for the proposed sensor, the affected transmission coefficient due to sample loading can be observed in Figure 7. The inverse squares of the resonant frequencies (*f*_r,1_ and *f* ʹ_r,1_) are extracted from the simulated transmission coefficient data and the results with the corresponding real permittivity (*ε*ʹ_r_) of the MUT are plotted and showed in Figure 7a,b.

In Figure 7a,b, it can be observed that the slope of the plotted curves relies on the thickness of MUT (*t_h_*) and increases as the thickness of the MUT increases. However, in Figure 7a, it can be observed that the slope of the curve residues roughly constant for the sample thickness (*t_h_*) larger than 9 mm. This specific behavior may be noticed from the two lines corresponding to the MUT thickness of 9 mm and 12 mm, where both curves overlap.

Moreover, in Figure 7a, it may be observed that all plotted curves corresponding to the MUT permittivity variation (*ε*ʹ_r_ = 2 to *ε*ʹ_r_ = 12) have a linear dependency, while in Figure 7b, all plotted curves corresponding to the MUT permittivity (*ε*ʹ_r_ > 10) have a roughly exponent dependency while the MUT thickness is increasing. In order to combine all the above effect, the dielectric constant of the specimen is mathematically expressed in terms of the family of straight lines as well as a family of exponential curves, where the freelance parameters are the resonant frequencies (*f*_r,1_ and *f*ʹ_r,1_ expressed directly in GHz) and the sample thickness (*t_h_* expressed directly in mm). By taking this aspect into consideration when using the fitting tool practically, the accuracy of the numerical model increases. So, based on the plotted data and using the curve fitting tool, we obtain the expressions for the real permittivity as a function of the MUT’s thickness and the two resonant frequencies:

(9) $$\varepsilon {’}_{r}=-\frac{1}{1.88\cdot 10^{-4}\cdot \ln(18472.96\cdot \ln(t_{h})}\cdot \ln\left[\frac{39.6824-{\left(f_{r,1}\right)}^{-2}}{39.095}\right]$$

(10) $$\varepsilon {’}_{r}=\exp\left\{2.2607\cdot \ln\left[\frac{{\left(f{’}_{r,1}\right)}^{2}-5.373}{\frac{0.2336}{t_{h}+0.1266}-1.2}\right]\right\}$$

These expressions will be used in our real-life measurements to determine which resonant frequency provides more accurate results. The mathematical limitation of relation (9) is for MUTs with thickness of 1 mm. In this case, relation (9) cannot be used, but we can use relation (10) instead. This case proves the limitation of the sensor, but also the importance of having an alternative analytical formula, based on the second resonant frequency.

### 3.2. Deduction of the Imaginary Part of the Permittivity

After finding the numerical relations (9) and (10) for determining the real part of the permittivity of the material under test, an identical analysis is completed to find a numerical relation for computing the loss tangent of the tested sample, which will give us information for determining the imaginary part of the permittivity.

The relation between the loss tangent, tan *δ*, the quality of the proposed sensor after loading the MUT, *Q*_MUT_, the real part of the permittivity, *ε*ʹ_r_, and the imaginary part of the permittivity, *ε*ʹʹ*_r_* is given by [32]:

(11) $$Q_{MUT}=\frac{1}{\tan\delta }=\frac{\varepsilon {’}_{r}}{\varepsilon ’{’}_{r}}$$

where *Q*_MUT_ states the quality factor of the proposed sensor after loading the MUT, which may be determined applying relation (7). The imaginary part of the complex permittivity is therefore obtained using (7) and (11).

**Figure 8:**
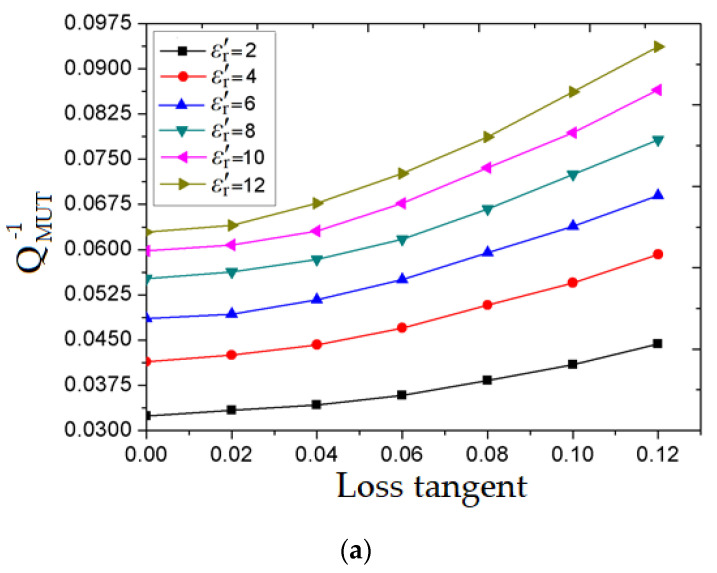

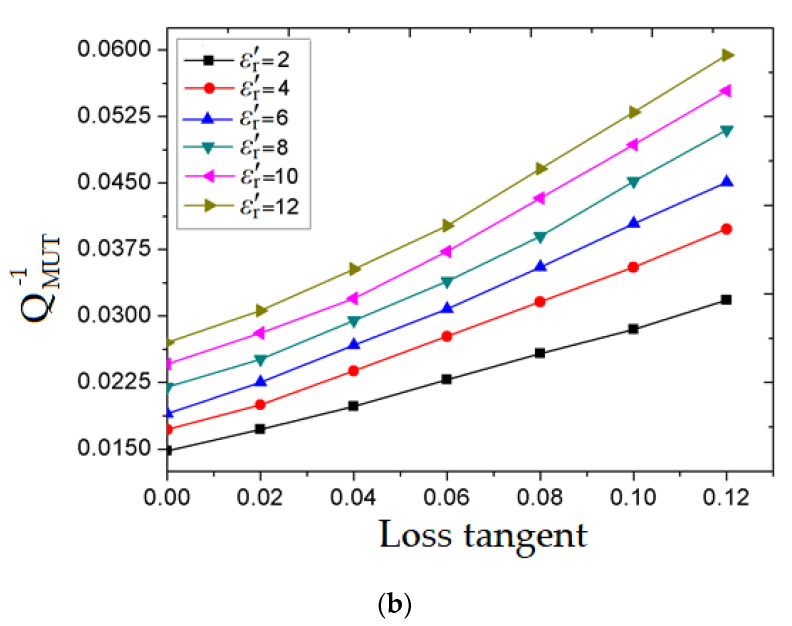
Inverse of Q-factor in terms of tan δ for various values of *ε*ʹ_r_, depending on resonant frequency: (**a**) *f*_r,1_; (**b**) *f ʹ*_r,1_.

At first, the real part of the permittivity takes values in the range of 2 to 12. For each value, the loss tangent is changed in the range from 0 to 0.12. For each change, the resonant frequencies (*f*_r,1_ and *f* ʹ_r,1_) are recorded from the S_21_ parameter’s simulation. Then, the quality factor is extracted from the simulation result of S_21_ as depicted earlier in Figure 5, for each resonance frequency. After that, the inverse of the quality factors for each corresponding loss tangent are plotted in Figure 8a,b.

In Figure 8a, it may be observed that all plotted curves corresponding to the MUT loss tangent variation (tan *δ* = 0 to tan *δ* = 0.12) have a semi-linear low slope component.

In Figure 8b, all plotted curves remain on a semi-linear high slope component. Therefore, to deduce the tan *δ* of the MUT, which relies on the loaded quality factor as well as the *ε*ʹ_r_ of the MUT, a curve fitting tool is utilized. As in the previous case of the real part of permittivity, a commercially available software OriginPro 8 is used to determine the numerical model for both extracted results in Figure 8a and b as presented in expressions (12) and (13), respectively:

(12) $$\tan\delta =\exp\left\{0.687\cdot \ln\left[8.165\cdot 10^{-3}\cdot \left(36.812\cdot \varepsilon {’}_{r}{}^{-0.338}-Q_{MUT}\right)\right]\right\}$$

(13) $$\tan\delta =0.1574\cdot \ln\left[\frac{Q_{MUT}^{-1}+0.0023}{0.04503-0.0318\cdot {\left(0.94427\right)}^{\varepsilon ’{’}_{r}}}\right]$$

After determining the *ε*ʹ_r_ from (9), (10) and tan *δ* from (12), (13) the imaginary part of the complex permittivity can be obtained using (11).

The mathematical limitation of relations (12) and (13) appear indirectly through the value of the real part of permittivity, *ε*ʹ_r._ If this quantity cannot be determined using the first resonant frequency, as in the case of 1 mm thick MUTs, then automatically neither the loss tangent using relation (12) can be determined. As in the case of real part of permittivity, a second option, one using the second resonant frequency is very useful in practical applications.

## 4. Results

The sensor proposed in Figure 4 is now implemented and measured. The substrate used is FR-4 (relative permittivity ε_r_ = 4.4 and the dissipation factor, tan δ, is approximately 0.02), with a thickness of 1.6 mm and cooper metallization electrodeposited on both sides of the substrate, with a thickness of 18 µm.

**Figure 9:**
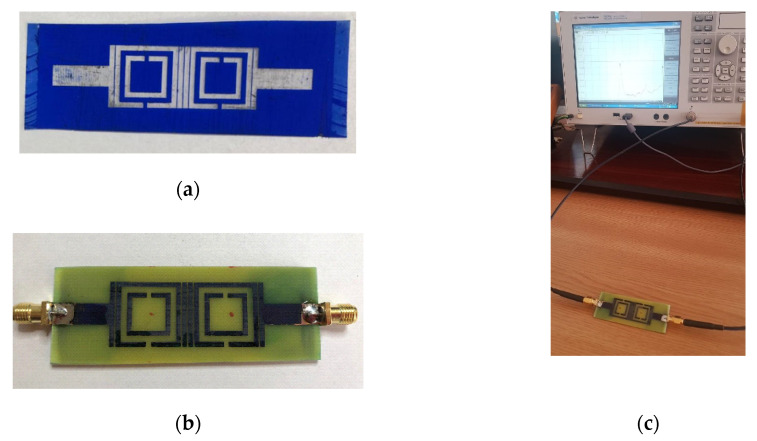
Implementation of the sensor: (**a**) Coated Mylar (Polyester) foil base material; (**b**) the sensor implemented on a FR-4 substrate, with 1.6 mm, with Cooper metallization on both sides; (**c**) measurement setup.

The technological development and manufacturing of the PCB sensor structure was made using Press-n-Peel Blue transfer foil from Techniks, with the etching process being done in turbulent and warm (approximately 50 °C) ferric chloride (FeCl_3_), with the concentration of 38%. Press-n-Peel method uses a coated Mylar (Polyester) foil base material in which several layers of release agents and resist coatings are applied, as shown in Figure 9a.

The width of the copper traces has been set to 1.52 mm, with the spacing between two concentric squares also being 1.52 mm, while the spacing between strips is 0.2 mm. The openings in the copper squares are equal to 1.22 mm. The ground plane size is 77 × 18 mm, being developed as a full copper plane onto the bottom side of the PCB. The overall dimensions of the sensor, access transmission lines included, is 76 mm × 26 mm, while the resonant structure itself, where the MUT is placed has a dimension of 44 mm × 18 mm.

The SMA (SubMiniature version A) connecters, which are classical semi-precision coaxial RF connectors used as interface for coaxial cables with screw-type coupling mechanism are mounted on the structure using mechanical welding. The SMA has a 50 Ω characteristic impedance and is designed to work in the range 0–18 GHz, fully matched with the necessities of the current sensing structure. The manufactured sensor is presented in Figure 9b.

The measurement setup consists of the sensor connected to the Agilent E5071C (9 kHz to 6.5 GHz) network analyzer through 50 Ω cables, as shown in Figure 9c. Before starting the measurements, a short-open-load-through (SOLT) calibration was performed using the Agilent calibration Kit. The number of sweep points is chosen 1601.

A set of materials under test: Transparent glass [32], acrylic glass [33], Teflon [32], and Plexiglas [34], with different thicknesses (*t_h_*) of 1 mm, 2 mm, 5 mm, and 10 mm is selected and used for measurements. For each measurement, the sensor is placed on a rough, stable surface and the MUT is carefully placed to cover the whole sensing area. Then, using the Agilent E5071C network analyzer, the magnitude of S_21_ parameter is measured. Further, it is inspected and, using a marker, the resonant frequencies and the relative 3 dB bandwidth of the resonator’s frequency response are read. Then, using relation (7), the quality factor of the loaded sensor is determined. The quality factor for the loaded sensor with real MUTs is determined based only on measurements. Both resonant frequencies obtained after measurement for different types of MUT are considered and using relations (9) and (10), two possible values for the real part of the permittivity are obtained. They are compared with reference values [32,33,34] and the results, including errors, are synthesized in Table 2.

Analyzing the data obtained after measurements, it can be observed that both resonant frequencies can be used to compute the real part of the permittivity, except for the case of 1 mm thickness Plexiglas MUT. In this case, relation (9) cannot be used, because of numerical limitations, but relation (10) gives a value, measured with an error less than 3.5% than the reference value, proving the importance of an extra resonant frequency. The best results are obtained for samples with thicknesses of 2 mm and 5 mm. Another important observation is that when using the second resonant frequency, the errors are slightly larger than those corresponding to using the first resonant frequency. This can be explained because of technological imperfections and placing the probe in direct contact with the sensor. Still, taken into consideration that the errors are quite small for both frequencies, smaller than 4%, it can be concluded that the sensor is suitable for accurate real part of permittivity measurements. The small errors show that the gap between the sensor and the MUT can be ignored.

For the measurement of the imaginary part of permittivity, first, the measured quality factor Q_MUT_ is replaced in relations (12) and (13) and the value of the loss tangent, tan *δ* is obtained. It is compared to the reference values [32,33,34] and the results are given in Table 3.

Analyzing the results in Table 3, we notice that for acrylic glass of 5 mm thickness, the error when using the second resonant frequency is smaller than for the first one. Overall, the measurements were done with less than 4% errors. Again, because we could not determine the real part of the permittivity for the first resonant frequency, we could not determine the loss tangent either. A good solution for such cases is to use the alternative, given by the second frequency.

Next, using relation (11), the imaginary part of the permittivity is determined. The results of the measurements are given in Table 4.

The errors in Table 4 are smaller when using the second resonant frequency, except for the transparent glass case, proving the importance of the second resonant frequency. Additionally, the errors determined in Table 4 show both the impact of approximations due to computing and the impact of measuring two parameters with different errors: The real part of permittivity and the loss tangent. So, we find cases when the errors are smaller than 1%, even if the corresponding errors for the loss tangent measurements alone are not that small. The observations regarding the technological imperfections and the placing procedure of the probe remain valid. Further, the impact of the air gap over the measurements was not considered and, still, the results are very good, much better than the ones in literature [1]. For example, for Teflon, we have obtained measurement errors of 1.19% and 3.91% for the real permittivity and 3.88% and 1.231% for the imaginary part, proving the accuracy of the results. In reference [1], the errors are 1.9% for the real part of permittivity and 8.6 2% for the imaginary part of permittivity. Nevertheless, it is worth observing that the thickness of the MUT has an impact on the overall response of the sensor. If the thickness of the MUT is increased, the interaction of the electromagnetic field is enhanced, so a change in the sensor’s frequency response is more obvious.

The results in the two tables show that the sensor can be used successfully to accurately characterize the dielectric parameters (dielectric constant and loss tangent) for both low-losses and lossy dielectrics, as well as for high dielectric constants dielectrics and small dielectric constants dielectrics.

## 5. Conclusions

In this paper, we present a modified SRRs planar sensor for noninvasive, accurate complex permittivity measurements of solid dielectrics. Starting from the classical SRR, a modified structure, using vertical strips added at a close distance of 0.2 mm to the SRR is investigated both from the enhanced selectivity perspective and from the overall dimensions. The result is a sensor made of two modified SRRs with lateral vertical strips, exhibiting high sensitivity for two resonant frequencies, at 1.24 GHz and 2.08 GHz.

A simplified equivalent circuit model is used to explain the microwave sensor’s design, and a very good agreement between the circuit model and the full electromagnetic simulation results is achieved. After a careful investigation, the two VS-SRRs sensor is selected to be further investigated. For each resonant frequency, we consider the data obtained after full wave electromagnetic simulation and using a curve fitting tool, we determine analytical expressions for both the real and imaginary parts of the permittivity. These are expressed as a function of the resonant frequencies, the MUT’s thickness, and the quality factor of the loaded sensor.

The sensor is implemented on an affordable, commercial substrate, FR-4 substrate, with a thickness of 1.6 mm, with reduced dimensions and being able to measure the real and imaginary parts of the permittivity for different solid dielectric samples, with errors less than 4.5% for both resonant frequencies in all analyzed cases. In our work, we have considered a large range of samples, with different thicknesses, different loss tangents, and dielectric constants to better investigate the sensor’s capabilities in real-life scenarios. The diversity of the samples helped us to observe the limitations of the numerical model developed in Section 3 and find solutions to overcome them, such as successfully using the second resonant frequency.

Also, we have measured the quality factor both for the unloaded and loaded sensor using the resonant frequency and the relative 3dB bandwidth of the resonator’s frequency response. This approach added more practical consistency to our investigation. Still, some improvements can be done with respect to further miniaturization and the possibility to use this sensor for liquid dielectric characterization.

In future, we will investigate if this sensor can be used to measure the permeability for magnetic samples and if a lower losses substrate will improve the results. Last, but not least, the modified structure will be investigated if it is suitable for other resonant applications, which require the usage of similar configurations.

## Figures and Tables

**Table 1:** Dimensions of the SRR in Figure 1a or Figure 2a.

Parameters	d [mm]	w [mm]	g [mm]	c [mm]	s [mm]
SRR	18.4	1.52	1.22	1.52	-
Proposed SRR	18.4	1.52	1.22	1.52	0.2

**Table 2:** Real part of the complex permittivity for different materials under test.

Material	t_h_ [mm]	ε’_r_	f_r1_ [GHz]	ε’_r1_	error [%]	f_r2_ [GHz]	ε’_r2_	error [%]
Transparent Glass	5	6	0.9856	5.872	2.12	1.671	6.163	2.72
Acrylic Glass	5	2.7	1.127	2.647	1.93	1.902	2.629	2.59
Acrylic Glass	2	2.7	1.139	2.644	2.04	1.924	2.627	2.7
Teflon	10	2.1	1.152	2.125	1.19	1.941	2.017	3.91
Plexiglas	1	2.597	1.155	-	-	1.97	2.512	3.24

**Table 3:** Loss tangent for different materials under test.

Material	t_h_ [mm]	tan δ	Q_1MUT_	tan δ_1_	error [%]	Q_2MUT_	tan δ_2_	error [%]
Transparent Glass	5	0.005	20.18	0.00512	2.39	47.39	0.0051	3.26
Acrylic Glass	5	0.02	26.12	0.019417	2.92	56.1	0.0203	1.88
Acrylic Glass	2	0.02	26.07	0.020532	2.66	56.05	0.0205	2.7
Teflon	10	0.0003	28.5317	0.000308	2.75	69.335	0.0003085	3.24
Plexiglas	1	0.0008	26.648	-	-	65.43	0.00082	3.12

**Table 4:** Imaginary part of the complex permittivity for different materials under test.

Material	t_h_ [mm]	ε’’_r_	ε’’_r1_	error [%]	ε’’_r2_	error [%]
Transparent Glass	5	0.03	0.030065	0.215467	0.031431	4.497
Acrylic Glass	5	0.054	0.051397	4.82074	0.053369	1.169
Acrylic Glass	2	0.054	0.054287	0.530756	0.053854	0.27129
Teflon	10	0.00063	0.000655	3.8888	0.00062	1.231
Plexiglas	1	0.002078	-	-	0.00206	0.8548
